# Opportunities for the application of advanced remotely-sensed data in ecological studies of terrestrial animal movement

**DOI:** 10.1186/s40462-015-0036-7

**Published:** 2015-05-04

**Authors:** Wiebke Neumann, Sebastian Martinuzzi, Anna B Estes, Anna M Pidgeon, Holger Dettki, Göran Ericsson, Volker C Radeloff

**Affiliations:** Department of Forest and Wildlife Ecology, University of Wisconsin-Madison, 1630 Linden Drive, Madison, WI 53706 USA; Department of Wildlife, Fish, and Environmental Studies, Swedish University of Agricultural Sciences, Umeå, SE-90183 Sweden; The Huck Institutes of the Life Sciences, Pennsylvania State University, Pennsylvania, USA

**Keywords:** Animal trajectories, Movement patterns, Remote sensing, Trade-off resolution, Satellite products, Landsat, LiDAR, MODIS, Animal movement databases

## Abstract

Animal movement patterns in space and time are a central aspect of animal ecology. Remotely-sensed environmental indices can play a key role in understanding movement patterns by providing contiguous, relatively fine-scale data that link animal movements to their environment. Still, implementation of newly available remotely-sensed data is often delayed in studies of animal movement, calling for a better flow of information to researchers less familiar with remotely-sensed data applications. Here, we reviewed the application of remotely-sensed environmental indices to infer movement patterns of animals in terrestrial systems in studies published between 2002 and 2013. Next, we introduced newly available remotely-sensed products, and discussed their opportunities for animal movement studies. Studies of coarse-scale movement mostly relied on satellite data representing plant phenology or climate and weather. Studies of small-scale movement frequently used land cover data based on Landsat imagery or aerial photographs. Greater documentation of the type and resolution of remotely-sensed products in ecological movement studies would enhance their usefulness. Recent advancements in remote sensing technology improve assessments of temporal dynamics of landscapes and the three-dimensional structures of habitats, enabling near real-time environmental assessment. Online movement databases that now integrate remotely-sensed data facilitate access to remotely-sensed products for movement ecologists. We recommend that animal movement studies incorporate remotely-sensed products that provide time series of environmental response variables. This would facilitate wildlife management and conservation efforts, as well as the predictive ability of movement analyses. Closer collaboration between ecologists and remote sensing experts could considerably alleviate the implementation gap. Ecologists should not expect that indices derived from remotely-sensed data will be directly analogous to field-collected data and need to critically consider which remotely-sensed product is best suited for a given analysis.

## Introduction

Both remote sensing and animal tracking technology have recently experienced major advances which has the potential to facilitate integrated analyses of environmental and animal movement data in unprecedented detail [1,2] Correspondingly, statistical analyses have advanced to quantify spatial patterns, to account for processes on different scales and for spatial autocorrelation in animal movement data [3]. Improvements in sensor tracking technology, e.g., Global Positioning System (GPS) data, provide animal movement data that capture movement paths (i.e., time series) in ecological landscapes, an improvement over “timeless” position clusters [1]. These advances provide many opportunities, but also challenge the scientific community of movement ecology as acknowledge with special issues in two leading international journals [1,2]. In parallel, remotely-sensed data from different satellite sensors have been available since the 1970s at a wide range of spatial and temporal resolutions, providing a better match to various scales of animal movement. A key challenge to overcome, however, is the time lag between the availability of new remotely-sensed products and their application in ecological research and management. Minimizing this temporal disconnection requires closer collaboration between wildlife ecologists and remote sensing experts to facilitate more rapid implementation of geospatial data processing (*sensu* [4]).

Analyses of animal movement pathways can increase understanding of resource utilization and dispersal dynamics among populations over time, and can ultimately aid animal conservation and management [5]. While habitat analyses tend to overlook the temporal aspects of habitat use, analyses of animal movement pathways (trajectories) integrate each animal location into the larger context of the spatial distribution of the population and changing environmental conditions through time, as well as individuals’ constraints, behavior, and survival [5-7]. Animal movement over time is a result of decision-making among behavioral trade-offs, considering animals’ internal state, motion, navigation, and external factors [8]. Today, sensor technology can link physiological data such as body temperature and heart rate to movement data, thereby directly linking animals’ physiological conditions and movement behavior to landscape features [9]. Movement path analyses (such as state-space models or step selection functions) track animal resource selection over time [10,11], and require increasing application of dynamic environmental covariates to better understand the mechanism for animal movement behavior.

Animals interact with their environment at multiple spatiotemporal scales, resulting in different movement modes [12,13]. Fine-scale temporal movement data reveal functional landscape connectivity by distinguishing movement corridors, barriers, or stop-over-sites, thereby helping to identity areas critical for mammalian and avian movement [14-18]. Fine-scale data also capture residency times and site fidelity, and can identify critical resources such as water or resting sites [19,20]. At broader scales, pathway analysis can distinguish between migration, nomadic behavior, and dispersal [21], which affects population dynamics, resource utilization, gene flow among populations (e.g. [5]), and the spread of diseases [22].

**Figure 1:**
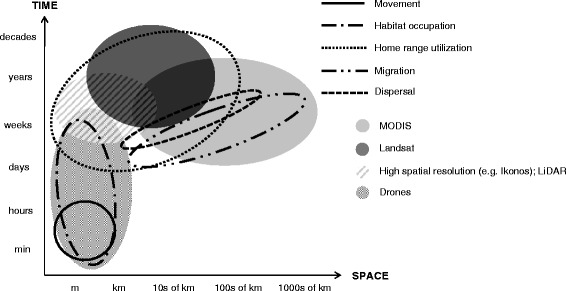
**Scales of current resolution in space and time of animal movement and remotely-sensed data.**

New remotely-sensed data better capture both the temporal dynamics of landscapes and the three-dimensional structure of habitats. In contrast to field-collected point data, which are typically information-rich but spatially-sparse, remotely-sensed data typically provide spatially continuous data over larger areas, but less information for any given point or pixel due to their relatively coarser resolution. This means that the different spatiotemporal scales at which animal movement occurs require different types of remotely-sensed data to understand the underlying causes of movement (Figure 1 [7,23]). However, there is an inherent trade-off between *fine-but-infrequent* (i.e., fine spatial but coarse temporal grain) versus *coarse-but-frequent* (i.e., coarse spatial but fine temporal grain) data recorded by different satellite sensors (Figure 1; Table 1). More specifically, if the goal is to understand fine-scale movement patterns, fine-but-infrequent data are arguably most useful, because they provide the detail necessary to gain insight into the drivers of movement patterns and because temporal variation of the environment such as land cover or phenology matters less over shorter time spans (see e.g. [13,24-28]). In contrast, to understand fine-scale movement patterns of flying animals, highly frequent information about weather and wind condition is needed because the fine details of e.g., atmospheric turbulence that shape the movement patterns can change very rapidly [29,30]. On the other hand, when broad-scale movement patterns are studied, temporal change in the environment is the key and *coarse-but-frequent* data are better covariates of movement (e.g. [31]).

**Table 1:** **Source and resolution of different remote sensing products**

**Sensor and satellite**	**Spatial resolution [m]**	**Temporal resolution [days]**	**Swath [km]**	**Operational since**	**Link**
MODIS (Terra, Aqua)	250, 500, 1000	1-2	2.330	1999 (Terra), 2002 (Aqua)	http://modis.gsfc.nasa.gov/about/design.php
AVHRR***	1100	<1	2.600	1981	http://noaasis.noaa.gov/
VIIRS (Suomi NPP)	750	1-2	3.000	2011	http://npp.gsfc.nasa.gov/
ASTER (Terra)	15, 30, 90	16	60	1999	http://asterweb.jpl.nasa.gov/
ETM+ (Landsat 7)	15, 30, 60	16	183	1999**	http://landsat.gsfc.nasa.gov
TM (Landsat 5)	30, 120	16	185	1984*	http://landsat.gsfc.nasa.gov
Vegetation 1 (SPOT 4)	10, 20	2-3	60	1998	http://www.astrium-geo.com
Vegetation 2 (SPOT 5)	5, 10, 20	2-3	60	2002	http://www.astrium-geo.com
Spot 6	1.5, 6	2	60	2012	http://www.astrium-geo.com
RapidEye satellites	5	5	25	2008	http://www.rapideye.com
IKONOS	0.8, 3.2	3	11	1999	https://www.digitalglobe.com
GeoEye-1	0.4, 1.7	3	15	2008	https://www.digitalglobe.com
QuickBird	0.6, 2.4	3	18	2001	http://www.digitalglobe.com
Worldview 1	0.5	2	18	2007	http://www.digitalglobe.com
Worldview 2	0.5	1	18	2009	http://www.digitalglobe.com

* The very first Landsat launch was in 1972, but is not longer in service. **due to an instrument failure all scenes after May 2003 have data gaps; ***different NOAA satellites. 1978 was a first attempt for AVHRR on a different satellite, which was improved and replaced in 1981 by the AVHRR sensor or the NOAA satellite. Additional missions are planned for 2013–2014, such as Landsat DCM (continuing the Landsat program with pixels sizes of 15, 30, and 100 m), Spot 7 (which combined with Spot 6 will provide satellite imagery at a temporal resolution of 1 day), and Worldview 3, with pixel sizes of 0.3 m and 1.2 m. LiDAR remote sensing data used in research applications are typically acquired from airborne systems, rather than from satellites as those described above. The LiDAR acquisitions specifications, such as laser pulse density and area cover, thus are flexible and depend on the objectives of the particular study. Detailed information about LiDAR data and specifications used in natural resource management are described in [122] and [123]. Aerial photos captured with drones have typically a high spatial resolution (e.g. <10 cm) and they cover small areas (~15-25 km; see [112] for an example of an inexpensive drone designed to monitor forests and biodiversity.

Our goal here was to provide a review for movement ecologists who are less familiar with the variety of remotely-sensed data available and their application in animal movement studies, reducing the present time lag in application of available remotely-sensed products in animal movement studies [4]. Thus, we first review the current applications of remotely-sensed ecological indices that deal with vertebrate movement patterns in terrestrial systems. Second, we introduce newly-available remotely-sensed products and discuss the opportunities that they provide for animal movement studies. We focused on studies that link animal locations and remotely-sensed data in terrestrial ecosystems, published between 2002 and 2013 (Table 2). Specifically, the studies we reviewed investigated how animals interact with their terrestrial environment over time, and are based on trajectories and range shifts by individuals and populations, thereby potentially aiding conservation and management. Current animal movement studies are heavily biased towards larger-bodied animals due to the weight and size of tracking devices. While there is a history of radio-tracking and linking large-bodied seabird movement to environmental features, it has only recently become possible to equip small-bodied terrestrial avian migrants with devices capable of tracking long-range movements [32,33]. Consequently, our review focuses largely on movement studies of large-bodied mammalian species.

**Table 2:** **Sample of ecological animal movement studies that apply remotely-sensed products**

**Land cover**	**Terrain**	**Infrastructure**	**Climate**	**Weather**	**Phenology/ Productivity**	**Species**	**Data origin (when available; multiple layers in bold)**	**Reference**
Small scale (e.g., daily movement, residence time, side fidelity, corridors)								
X	X					Woodland caribou	LANDSAT, DEM^a^	[12]
X	X	X		X		Elk	**Snow water equivalents**, DEM^w^, roads^w^, habitat^w^	[124]
X	X	X				Bison	Vegetation cover type, geothermal layers^b^, DEM	[44]
X	X	X				Roe deer	SPOT, DEM^c^, digitized aerial photographs	[50]
X		X				Elk	**LANDSAT** ^v^	[34]
X		X		X	X	African elephant	**LANDSAT**, TRMM, **MODIS** (EVI)	[35]
X	X					Moose	Ecoforest maps, DEM	[13]
X	X	X				Brown bear	CORINE, DEM^u^, human features^u^	[42]
X	X	X				Gray wolf	LANDSAT^d^, DEM^e^	[39]
X						North Island robin	digitized aerial photographs, satellite images	[24]
X	X	X				Grizzly bear	LANDSAT^f^, vegetation inventory data, fire-history maps, DEM	[51]
X						Lion	digitized water wholes	[19]
X	X	X			X	African elephant	digitized static features, DEM^g^, SPOT (NDVI)	[40]
		X			X	Jaguar	Roads^h^, **MODIS** ^c^ (NDVI)	[125]
X		X				American marten	**color-infrared aerial photographs**	[15]
X						Barred Antshrikes, Rufous-naped Wrens	infrared images^g^, orthorectified using DEM	[126]
X		X				Black bear	LANDSAT, color orthophotos, stream water layer^c^, topographic maps	[52]
					X	Mule deer	**MODIS** ^m^ (NDVI)	[16]
X						Lion	LiDAR^s^	[110]
Coarse/seasonal scale (e.g., seasonal range change, migration, dispersal)								
	X				X	Wildebeest	DEM, GTOPO30^c^, **AVHRR/NOAA** (NDVI)	[127]
					X	Red deer	**NOAA** (NDVI)	[128]
	X		X		X	Serengeti Wildebeest	DEM, SRTM, LANDSAT, **NOAA**, **SPOT** (NDVI)	[58]
					X	Elk	**MODIS** (NDVI)	[66]
					X	Five migratory bird species^j,k^	**AVHRR/NOAA** (NDVI)	[60]
					X	Mongolian gazelle	MODIS (NDVI)	[129]
	X		X		X	Saiga antelope	DEM, SRTM^i^, **MODIS** (NDVI); **WorldClim database** (precipitation)	[68]
X					X	Migratory birds	**LANDSAT**	[14]
X	X		X			Roe deer	EEA-Corine Land cover, CGIAR-DEM/SRTM, NASA-ASTER relative DEM, **MODIS** (snow)	[38]
				X		Great snipes	**NCEP/NCAR** provided by NOAA/OAR/ESRL PSD^r^ (wind)	[30]
					X	Four migratory ungulate species^l^	**AVHRR/NOAA**, **GIMMS** (NDVI)	[31]
	X					Red deer	DEM^t^	[130]
	X			X		Golden Eagle, Turkey vulture	DEM, GTOPO30, **NARR** ^o^ (wind, temperature)	[29]
				X		Common swift	**NCEP/NCAR** provided by NOAA/OAR/ESRL PSD^r^ (wind)	[17]
X		X	X		X	African buffalo	MODIS (EVI, tree cover^p^), TRMM^n^, **MODIS** ^o^ (fire), vegetation map^q^	[41]
X			X		X	Bobolink	**MODIS** (NDVI), **SPOT** ^x^, **water content estimates** ^y^	[59]
Small and coarse scale								
					X	African elephant	**AVHRR/NOAA**, **SPOT** (NDVI)	[61]
					X	Sheep	**MODIS** (NDVI)	[67]

LAND COVER: vegetation, water, streams, forest age, cutblocks, seral stages; TERRAIN: elevation, slope, aspect, ruggedness, solar radiation, soil wetness; INFRASTRUCTURE: roads, buildings, borders, fences, trails; CLIMATE/WEATHER: wind speed, wind direction, precipitation, temperature, cloud; PHENOLOGY/PRODUCTIVITY: NDVI, EVI, tree cover, fire; ^a^British Columbia Ministry of Crown Lands; ^b^The Watershed Institute (California State University, Monterey Bay, USA), National Hydrographic Dataset; ^c^USGS; ^d^Foothills Research Institute Grizzly Bear Research Program; ^e^Banff and Kootenay National Park; ^f^Alberta Vegetation Inventory; ^g^NASA, CARTA program; ^h^Selva Maya Zoque y Olmeca database; ^i^Surface Radar Topography Mission; ^j^Ringing data; ^k^Lesser Whitethroat*,* Whitethroat*,* Blackcap*,* Chiffchaff, Willow Warbler; ^l^barren-ground caribou, Mongolian gazelle, guanacos, moose; ^m^Wyoming View; ^n^ http://trmm.gsfc.nasa.gov; ^o^National Deneter for Environ Predict; ^p^Wegmann *et al.*, unpublished data; ^q^Mendelsohn 1997, An environmental profile and atlas of Caprivi. Windhoek, Namibia: Gamsberg Macmilian; ^r^Boulder, Colorado, USA, http://www.cdc.noaa.gov; ^s^Carnegie Airborne Observatory, ^t^National Mapping Agency of Norway, ^u^Lantmäteriet Sweden, ^v^Alberta Sustainable Resource Development, ^w^Spatial Analysis Center at Yellowstone National Park, ^x^ http://bioval.jrc.ec.europa.eu/products/glc2000/products.php, ^y^ http://neo.sci.gsfc.nasa.gov.

## Review

### Remotely-sensed products commonly used in ecological animal movement studies

The studies of small-scale movement that we reviewed included data about land cover, infrastructure, and terrain, and less often data on vegetation phenology, climate and weather (Table 2). Surprisingly, we found that even long-term, small-scale movement studies seldom applied multiple years of land cover data, although the satellite data from which they are derived, i.e. Landsat imagery, are available continuously since the 1970s (but see [15,34,35]). Moving beyond a single-date snap-shot of a given environmental situation towards incorporation of multiple years of data could help to detect land use change [36] which influences animal movement. Studies that analyzed animal movement at coarser scales (e.g., migration) often used data on vegetation phenology (e.g., the Normalized Difference Vegetation Index (NDVI)), sometimes combined with climate, weather, and terrain data (Table 2). While the use of land cover data from multiple dates was rare, phenology was typically included from multiple dates (Table 2). Unfortunately, we also found that ecological studies frequently lacked details regarding the origin of the satellite data, and the spatial and temporal resolution of the products that had been analyzed (e.g., land cover data; Table 2). This lack of information makes it difficult to determine, for example, if the resolution of the remotely-sensed products matches the resolution of the animal location data and is suitable to address the study question, ultimately impeding the readers’ interpretation of results [18].

*Land cover* data are typically derived from *fine-but-infrequent* remotely-sensed products and researchers can use them to infer animal movement patterns on finer spatial scales (Figure 1). To suggest appropriate conservation actions based on studies of movement, it is important to relate animal movement to land cover and land use, and to understand animals’ use of the landscape, such as, the reluctance of dispersing North Island robins *Petroica longipes* to cross pastures, or elephants’ *Loxodonta africana* need to be close to water resources during the dry season [24,35]). Ancillary data can capture land use change and infrastructure development and can help to update remotely-sensed maps, thereby creating dynamic landscape data [15]. Of the studies we reviewed, Landsat or aerial photographs were the most common source. Images provided by Google Earth (i.e., satellite data from Landsat, IKONOS, and Quickbird) provide a visualization tool, and have been used to understand habitat use during avian migration for Veery *Catharus fuscescens* [37], but are not suitable for analyses requiring image manipulation.

*Digital elevation models* (DEM) provide *fine-but-infrequent* information on altitude, aspect, and slope, as well as soil wetness and solar radiation, and have been applied in different ways. DEMs are sometimes combined with *coarse-but-frequent* remotely-sensed atmospheric variables such as wind speed, turbulent kinetic energy, temperature, and cloud height (e.g., 8-day moderate-resolution Imaging Spectroradiometer (MODIS) products, monthly WorldClim data, or 10-day National Oceanic and Atmospheric Administration data (NOAA; i.e., the Advanced Very High Resolution Radiometer (AVHRR); Table 2). DEMs can be a powerful tool and the interpretation of observed patterns can contribute to inference of migratory strategies, for example disentangling species-specific specialization to uplift modes in soaring birds such as Turkey vultures *Cathartes aura* and Golden Eagles *Aquila chrysaetos* [29]. The analysis of digital elevation and climatic data in relation to animal movement data highlights that topographic variability and winter severity affect migratory behavior in ungulates such as roe deer *Capreolus capreolus*, and their opportunistic migration behavior when there are no predictably extreme winter conditions [38].

*Human infrastructure* influences many wildlife species’ habitats. Infrastructure can create barriers and can modify daily and seasonal movement patterns. For example, gray wolves *Canis lupus* cross highways less frequently with increasing human presence [39], human settlements disrupt African elephant *Loxodonta africana* movement [40], and distance to fences affects African buffalo *Syncerus caffer* migrations [41]. In hunted species, infrastructure can also affect temporal movement patterns, e.g., brown bear *Ursus arctos* and moose *Alces alces* avoid roads during the day [42,43]. On the other hand, some species associate preferentially with infrastructure, for example to facilitate movement, as in the case of the American bison *Bison bison* [44]. Digital maps of infrastructure are *fine-but-infrequent* data, and typically derived from land cover data and aerial photos. The use of high spatial resolution imagery and LiDAR (Light Detection and Ranging; pulsed laser signals that measure distances to the earth surface and thereby generate information about the shape of the surface; http://oceanservice.noaa.gov/facts/lidar.html) also provides opportunities to discriminate between different types of human infrastructure and vegetation, such as highways, roads, railroads, pipelines, power-lines, houses, and trees (e.g. [45-48]). In high-resolution satellite imagery, the number of lanes of a given road is easily determined, and LiDAR data can detect roads even under dense forest canopies [49]. Governments and private companies use LiDAR and high spatial resolution data to map human infrastructure for large areas, and the resulting maps can be useful for understanding wildlife movement. Understanding road-crossings, the impact of road density and movement barriers such as wildlife fences, and exploitation or avoidance of road-near habitats, provides insights important for wildlife conservation and management (e.g., elk *Cervus elaphus* [34]; African elephant [35]; roe deer [50]; Grizzly bear *Ursus arctos* [51]; Black bear *Ursus americanus* [52]).

*Plant phenology*, the annual dynamics of vegetation greenness, can be tracked using NDVI and the Enhanced Vegetation Index (EVI) [53,54], although NDVI is more commonly applied in animal movement studies (see review by [55]). Phenology indices are typically derived from *coarse-but-frequent* satellite imagery, such as 10-day Satellite Pour l’Oservation de la Terre (SPOT) or 16-day MODIS reflectance data, and can be applied to predict individual and population movement and distribution. Yet animal movement ecologists need to consider the different interpretation of traditional phenology and satellite-based landscape phenology; while traditional phenology tracks e.g. flowering and budding of single plant species, satellite-based phenology quantifies dates of greening and browning patterns of multiple plant species at the landscape scale [56,57]. The analysis of variation in broad-scale landscape predictability is useful for gaining insights into long-distance movements in ungulates such as migration, nomadism, and residency [31]. Vegetation proxies such as NDVI also provide opportunities for applications in environmental conservation and paleoecology, and for predictions of past and future population and biodiversity response to climate, phenology, and primary production [55]. In our sample of studies, plant phenology data were commonly used to understand broad-scale migration patterns. For example, new forage growth is important for ungulate and avian long-distance migration (e.g., wildebeest *Connochaetes taurinus* [58]; Bobolink *Dolichonyx oryzivorus* [59]), and environmental conditions en route affect arrival at the breeding grounds of migratory bird species [60]. NDVI is particularly useful for movement studies in highly seasonal ecosystems (e.g., African elephants move more randomly when forage is abundant [61]). The occurrence of fires affects plant phenology and modifies animal movement (e.g., African buffalo [41]). MODIS fire detection data are available from 2001 to present, and may facilitate reconstruction of prior habitat conditions [62]. The combination of vegetation indices (e.g., NDVI) with precipitation data (e.g., TRMM (Tropical Rainfall Measuring Mission)) improves the prediction of the timing and speed of migrations (e.g., zebra *Equus burchelli antiquorum* [63]). For vertical migration, i.e., along an elevational gradient, vegetation proxies combined with DEM can predict seasonal movement that follows vegetation growth at different elevation (e.g., for elephants [64]). For birds, combination of NDVI and weather data such as wind and temperature generates fruitful environmental indices helping to understand global long-term movement patterns (e.g., vultures [65]). Furthermore, new products, such as global data on soil moisture (SMAP, Soil Moisture active Passive, newly launched, http://smap.jpl.nasa.gov/) and the Global Precipitation Measurement (GPM, planned for the near future, http://www.nasa.gov/mission_pages/GPM/main/#.VNtBJy7s7FQ) provide exciting opportunities to improve our understanding of the drivers of animal movement.

*Productivity*, i.e., seasonal plant growth, can be calculated by merging information from ground biomass measurements and MODIS-derived NDVI, and can predict, e.g., movement patterns of elk at different scales, demonstrating the connection between migration and access to higher-quality forage [66]. With its temporal resolution of typically 10–16 days, NDVI has been used to infer fine-scale movement patterns related to vegetation greenness, where it may represent a static habitat index rather than a phenological index (e.g., forage availability does not affect within-patch movement in sheep *Ovis aries* [67], but affects migration speed across patches (i.e., stop-over-sites) in mule deer *Odocoileus hemionus* [16]).

*Climate and weather indices* provide information about precipitation (i.e., rainfall and snow from 10-day NOAA, 8-day MODIS, and monthly WorldClim data) and temperature (e.g., 10-day NOAA), and are generally coarse-but-frequent indices. It is important to distinct between climate and weather. *Climate* describes the average pattern of meteorological variables such as temperature, wind, precipitation over longer time in a given area. In contrast, *weather* describes the short-term variations of those meteorological variables. In the context of animal movement, climate affects animals at a broader scale and can, for example, cause seasonal migration. Precipitation and temperature are particularly important determinants of animal movement patterns in seasonal ecosystems (e.g., rainfall and snow influence migration patterns in Saiga antelope *Saiga tatarica tatarica* [68] and roe deer [38]; Table 2). *Weather*, on the other hand, affects short-term movement such as daily movement. In flying animals, dynamics in diurnal weather conditions generate complex environmental conditions that affect animals’ movement patterns (e.g., thermal conditions generate inter-specific migration strategies and movement behavior in soaring birds [29,69-71], wind drift such as tail- or crosswinds affect flying patterns in fruit bats *Eidolon helvum* [72], and temperature and turbulent kinetic energy affect bee-eater *Merops apiaster* flight mode [73]. *Climate and weather indices* provide information about precipitation (i.e., rainfall and snow from 10-day NOAA, 8-day MODIS, and monthly WorldClim data) and temperature (e.g., 10-day NOAA), and are generally *coarse-but-frequent* indices.

In summary, inclusion of remotely-sensed data can greatly improve our understanding of the drivers of animal movement and provide novel insights about movement strategies across species and ecosystems. The main environmental indices are available from a broad range of remotely-sensed products that can vary in precision, and accuracy (Table 3). The set of studies that we reviewed reflected the current application of remotely-sensed data in animal movement studies. Single-date Landsat imagery was a common source of land cover information. Terrain data combined with climate and weather data were powerful tools for inferring migration patterns. Yet, movement ecologists should critically weigh which remotely-sensed product best fits their scale of analysis (i.e., *fine-but-infrequent* versus *coarse-but-frequent*), and more importantly, should avoid the expectation that remotely-sensed data are equivalent to field-collected data. On the other hand, field-collected data will never match the spatial continuity of remotely-sensed data collected over large areas. Remotely-sensed and field-collected data have unique strengths, and their integration allows unique insights.

**Table 3:** **Precision and accuracy of main remotely-sensed derived products**

**Product**	**Sources**	**Precision**	**Accuracy**
Land cover	NLCD (National Land Cover Database): developed from Landsat 30-m pixel; available for 1992, 2001, 2006, and 2011. U.S. only.	20 classes	78 and 79% for latest products [117]
	CORINE: developed from Landsat, Spot, and recently higher resolution imagery; available for 1990, 2000, 2006, 2012. Minimum mapping unit/with 25 ha/ 100 m.	44 classes	8% (for 2000 version) [131]
	MODIS Land Cover: available for 2001–2012, 500-m pixel resolution	17 classes	75% [132]
	GlobCover: based on 300-m pixel resolution imagery from MERIS sensor (ENVISAT), available for 2005–06 and 2009.	23 classes	58-79% [133]
Terrain (elevation)	SRTM (Shuttle Radar Topography Mission): 30-m and 90-m pixel resolution	16-bit	Geolocation error = 9.8 meters; absolute height error = 6.9 meters; relative height error = 7.0 meters [134]
	ASTER Global Digital Evalation Map: 30-m pixel	16-bit	Overall accuracy ~17 meters [135]
	TanDem-X (TerraSAR-X add-on for Digital Elevation Measurement): provides elevation data <12-m pixel resolution; launched in 2010.	16-bit	<2 m height accuracy [136]
Climate/Weather	MODIS Land Surface Temperature/Emissivity: available daily, 8-day, and monthly, at 1-km, 5.6-km resolutions.	8-bit and 16-bit	0.5 K to 1 K [132]
	TRMM (Tropical Rainfall Measuring Mission): 16 times per day, multiple products describing rainfall at 2.4 km and 5-km pixel resolution	na	TRMM Precipitation Radar instrument is able to detect fairly light rain rates down to about .0.7 mm per hour.
	MODIS Cloud Product: daily product of cloud properties at 1-km and 5-km pixel resolution.	na	na
Phenology	MODIS Global Vegetation Phenology product (MCD12Q2): provides estimates of the timing of vegetation phenology at 500-m pixel resolution. MCD12Q2 is produced once a year with 24 months of input data, available from 2001 through 2012	16-bit	Consistent with in-situ measurements [137]
	SPOT Vegetation: provides NDVI global data since 1998 at a 1.15-km pixel resolution	na	na
	MODIS Gross Primary Productivity (GPP) product (MOD17A2): 8-day composite at 1-km spatial resolution	8-bit and 16-bit	Annual estimates of GPP are within 10.4% of average published results [132]
	MODIS Leaf Area Index (LAI) and Fractional Photosynthetically Active Radiation (FPAR): 8-day composite at 1-km spatial resolution	8-bit	Accuracy is 0.66 LAI units RMSE and 0.12 FPAR units RMSE respectively [132]

Note: The Visible Infrared Imaging Radiometer Suite (VIIRS) extends and improves the measurements initiated by the Advanced Very High Resolution Radiometer (AVHRR) and the Moderate Resolution Imaging Spectroradiometer (MODIS). na: no information.

### Opportunities for remotely-sensed products in ecological studies of animal movement

#### Increasing access to remotely-sensed data

High spatial resolution imagery is recorded by sensors such as IKONOS, QuickBird, OrbView-3 and Spot-5, with spatial resolutions from 0.5 - 10 m ([74]; Table 1). High-resolution imagery allows the identification of small habitat features such as infrastructure (e.g. [75]), riparian corridors (e.g. [76,77]), and individual trees (e.g. [78]). Knowledge of specific environmental features is often critical for understanding animals’ response to landscapes (see [79]), and improves our insight into species’ ecology (e.g. [20,50]). Although the cost of high spatial resolution imagery can be a limitation, cost has declined as more sensors have launched and increased competition [74]. Furthermore, in many areas high-spatial resolution images are available for free, thanks to web-mapping sites such as Google Earth (http://earth.google.com).

However, high-resolution imagery is not available everywhere and imagery archives are limited. In contrast, Landsat satellite imagery is available for free, and archives span 40 years, making Landsat the most commonly used satellite imagery for monitoring land cover [80], and in ecological studies (e.g. [81,82]; Table 1). The launch of the Landsat 8 satellite in February 2013 ([83]; Table 1), together with the planned Sentinel satellites of the European Space Agency [84], promises a new era in which 30-m resolution Landsat-like satellite data are available every two to three days, i.e., with similar temporal resolution as currently available for 500–1,000 m resolution MODIS and VIIRS.

Raw reflectance data (i.e., unclassified data), as an alternative to traditional classified land cover data or calculated indices such as NDVI, experience increasing interest for predicting animal distribution. They overcome classification errors and can provide better predictive ability – even at fine resolution – than more commonly-used remotely-sensed products (see for example in birds [85]).

A computational challenge for many movement ecologists is the integration of these remotely-sensed data sets into animal movement data sets, as remote sensing data often are provided using complex tiling systems in space and time [86]. Further, these data sets often vary in source, format, and projection system, making it difficult if not impossible for anyone others than remote sensing experts to make use of directly. However, new tools are emerging for movement ecologists which greatly automate and simplify the computation and integration [86]. In movement data portals, linking of environmental data to animal movement data is a general trend, making a variety of remotely-sensed products readily available for animal movement ecologists; e.g. through the *Env*-DATA system in *Movebank* ([86]; https://www.movebank.org), as a direct link in *Eurodeer* ([38]; www.eurodeer.org), or under development in *Wireless Remote Animal Monitoring* ([87]; *WRAM*).

#### Capturing the temporal dynamics of landscapes

The use of high temporal resolution satellite data offers new possibilities for animal movement studies as it means increased opportunities for matching satellite imagery with concurrent high resolution animal location data, and captures many temporal dynamics of landscapes (e.g., phenology, land use change, disturbances). In particular, long-term animal monitoring projects can take advantage of analyzing movement patterns in dynamic landscapes to better understand the impact of a given change and its consequence for management and conservation [88,89].

For studying fine-scale animal movement, an exciting advancement is the automated analysis of dense time series of Landsat images. In the past, studies rarely incorporated more than a few Landsat images due to cost and processing limitations. Since Landsat data became freely available in 2008, new image processing algorithms have been developed that analyze many decades of annual Landsat imagery simultaneously [90,91]. The great advantage of such dense time series (or ‘time stacks’) is in identifying changes, such as forest clear-cuts that can be detected immediately after the disturbance occurs, but can be missed if images are years apart. Software that analyses forest disturbance from dense time series of LANDSAT imagery includes Vegetation Change Tracker (VCT [90]) and LandTrendr [91]. Dense time series have also been used to monitor desertification [92] and urban development ([93]; Table 1).

For analysis of coarse-scale animal movement, indices of vegetation phenology can be informative [55]. Satellite sensors such as MODIS, AVHRR, VEGETATION, and the recently launched VIIRS provide daily imagery, typically summarized in 8- or 16-day composites that are relatively cloud-free (Table 1). However, composites may contain some erroneous NDVI values, and analyzing dozens of satellite images for a given year is computationally and logistically challenging. Thus algorithms that fit phenology curves to a time series of NDVI data and estimate parameters such as the onset of greenness, or the range of NDVI for each pixel are a major advancement [94,95]. By fitting a curve, the effects of outliers are reduced, reduce data volumes of phenology indices, and offer ecologically meaningful measures of vegetation change over time. One software package for phenology analysis is TIMESAT (http://www.nateko.lu.se/TIMESAT; version 3.0 [94]); MODIS phenology products are also available (https://lpdaac.usgs.gov/products/modis_products_table/). Substantial work has been done to unify data from older and new sensors to establish consistency in time series data [96] and improve phenological assessment through Landsat-MODIS fusion [97].

Although NDVI is the most commonly used index in ecological applications of remotely-sensed data, there is also a broad range of spectral indices that can be derived from satellite data (e.g. chlorophyll, foliage, surface moisture [98,99]) as well as ready-to-use products (e.g. Gross and Net Primary Productivity from MODIS) that offer opportunities for animal movement studies. The spectral fidelity (the number of spectral bands, and bandwidths) of remotely-sensed data is becoming increasingly important, as it is now possible to map individual plant species and nitrogen content of grazing lands. For example, RapidEye sensors provide high-resolution satellite images in which the Red-Edge band is sensitive to chlorophyll content and leaf structure (http://blackbridge.com/rapideye/). RapidEye data are at the cutting-edge of geospatial data, because of their ability to describe foliar nitrogen at impressive resolutions [100], which can help to understand herbivore distribution in grazing systems.

#### Characterizing the 3d strcuture of habitats

The 3D structural characteristics of vegetation are important determinants of animals’ space use [4]. For example, visibility at ground level and canopy cover affect selection of daytime bedding sites (e.g., brown bears [20]). Unfortunately, remotely-sensed products commonly used in animal movement studies, i.e., Landsat and MODIS imagery, are not well suited to quantify vegetation structure, resulting in a resolution gap between the movement data and the remotely-sensed vegetation indices.

LiDAR is a relatively new technology that uses a laser to measure the vertical and horizontal structure of ecosystems ([101]; Table 1), making it possible to characterize animal habitats in novel ways [102]. LiDAR data provide direct information about vegetation height [103] and canopy closure [104], and indirect information about forest age [105], canopy gaps [106], and large trees [107]. The ability of LiDAR to “see through” the forest canopy allows the characterization of topography and understory vegetation, important to many animal species [108]. For these reasons, LiDAR data are great predictors of species habitat use [102], and can track herbivore activity e.g., treefalls by elephants [109]. However, their utility for movement pathway studies still needs to be fully explored (but see [110] for lion *Panthera leo* hunting strategies and for predation risk in roe deer [111]). Most LiDAR data stem from airborne platforms, and are thus costly to obtain. As a result, the application of LiDAR data in ecological movement studies will likely depend on available LiDAR data from other projects for the foreseeable future. However, as more LiDAR platforms become available, costs are likely to decrease, and accessibility of LiDAR products will increase.

#### Environemntal real time assessment

Finally, scientists and managers are exploring the use of low-cost pre-programmed or remote-controlled drones equipped with video and photo cameras, as alternative ways to survey landscapes, count animal populations, and monitor human activities (see http://www.fort.usgs.gov/RavenA; e.g. [112]). This environmental assessment in real-time provides exciting opportunities for animal movement studies. Weather surveillance radars track arrival of migratory birds and thereby assess quantitative data on bird distribution, helping to identify migratory stopover over broad geographic areas in real-time [14,113].

#### Model reanalysis products

In addition to the technologies and products outlined before, there is a growing number of efforts to reanalyze remote sensing and weather data to derive consistent, long-term, global or regional environmental products at low to medium (12–1000 km) spatial resolution, but very high (1–24 h) temporal resolution. Examples include the North American Regional Reanalysis (NARR), the NOAA global weather reanalysis (NCEP Reanalysis 2), and the European weather reanalysis (ECMWF) for weather data, or the Oregon University Ocean NPP reanalysis for ocean products. The NASA’s MERRA project, for example, provides consistent information on weather variability from 1979 to the present. Finally, although not a reanalysis product per se, Hansen and co-authors [114], recently mapped global tree cover at yearly time steps between 2000 and 2012 at a spatial resolution of 30 meters, with great potential for animal movement studies.

### Potential limitations of remotely-sensed products for studies of movement ecology

As much promise as new remotely-sensed data and methods offer, it is important to recognize inherent limitations of these approaches. One important issue is that of errors, uncertainties, and accuracies in remotely-sensed products [115,116]. No land cover classification is perfect, and this can introduce errors in models of animal movement. Closely related to the question of accuracy is the question of the thematic resolution, i.e., the number of land cover classes, which for ecological applications often needs to be high. However, the classification accuracy decreases as more land cover classes are mapped. In the 2006 National Land Cover Dataset (NLCD), for example, forest as a single class has a user accuracy of 93%, but coniferous, deciduous and mixed forests each had accuracies of 81-85% [117]. Some land cover classes are more challenging to map than others; in the NLCD, for example, wetlands user accuracy was <40%. Spectral indices such as NDVI, on the other hand, are mathematical formulas and as such do not have a classification error, but require validation due to e.g. sensor errors or atmospheric contamination [118,119].

Movement ecologists that apply remotely-sensed products to animal movement data also need to consider spatial autocorrelation that are inherent in the data. Advances in statistical tools help to discover spatial patterns and to correct for autocorrelated data, e.g., in mixed models with help of correlation structures [3,120]. Patterns of autocorrelation in animal movement behavior themselves provide valuable information, and are used to better understand the mechanisms of animal movement [121].

The spatial resolution of the satellite data is another critical issue that is pertinent when analyzing fine-scale movement data. First, the size of the pixels, which tend to be 250–1,000 m for those satellites that provide daily observations, are far coarser than GPS-collar-based movement data. Secondly, the smallest observable feature that can reliably be mapped (i.e., the minimum mapping unit) is considerably larger than the pixel size (typically at least four pixels). This means that only Quickbird or Worldview data can detect habitat features of 1–5 m^2^, compared to minimum mapping units of 3,600 m^2^ for Landsat imagery, and 250,000 m^2^ for MODIS data. As movement ecology advances, and integrates more remotely-sensed data, it will be important to keep these inherent limitations of satellite data in mind, and to avoid the temptation to try to obtain more information from the imagery than it contains.

## Conclusions

Animals move in response to environmental changes, and to fulfill their varying needs for environmental resources over days, weeks, and years. New generations of remotely-sensed products with better spatiotemporal resolution provide great opportunities for insights into animal movement, as they characterize environmental patterns such as temporal dynamics of landscapes and three-dimensional structures of habitats. To capture these dynamics and to improve predictive ability, we recommend that researchers analyze time series of remotely-sensed products, as they can capture environmental dynamics that determine movement responses. The integration of landscape dynamism in movement studies becomes particularly important with respect to meeting the conservation and management challenges of future landscape change and corresponding changes in animal movement in space and time. To be able to provide realistic foundations for management and conservation, movement ecologists need to increase their application of dynamic environmental information when analyzing animal movement. With new tools emerging to automate the integration of remotely-sensed data with animal movement data sets, former computational challenges can be overcome, making environmental data more easily accessible for movement ecologists. Yet, the increasing possibilities of integrated analyses of remotely-sensed products and animal movement data presents new challenges to match these types of data logically, bearing in mind the inherent trade-offs posed by the different resolutions. It is important to critically weigh which remotely-sensed product is best suited for a given scale of analyses (e.g., *fine-but-infrequent* versus *coarse-but-frequent* or minimum mapping unit) and how its limitations may affect inferences. Closer collaboration between ecologists and remote sensing experts can facilitate the use of newly available products in movement studies, and minimize the time lag of applying these products in ecological research.

## Abbreviations

AVHRR: Advanced very high resolution radar
DEM: Digital elevation model
EVI: Enhanced vegetation index
GPS: Global positioning system
LiDAR: Light dectection and ranging
MODIS: Moderate resolution imaging spectroradiometer
NLCD: National land cover dataset
NDVI: Normalized difference vegetation index
NOAA: National oceanic and atmospheric administration data
SPOT: Satellite pour l’Observation de la Terre
TRMM: Tropical rainfall measuring mission
